# A conceptual exploration on the synergistic anti-tumor effects of high-order combination of OHSV2-DSTE^FAP5/CD3^, CAR-T cells, and immunotoxins in hepatocellular carcinoma

**DOI:** 10.3389/fimmu.2025.1509087

**Published:** 2025-05-08

**Authors:** Shuang Dong, Xin Chen, Xiaoyu Li, Yang Wang, Qing Huang, Yuanxiang Li, Jing Jin, Xianmin Zhu, Yi Zhong, Qian Cai, Chang Xue, Fang Guo, Le Huang, Mingqian Feng, Binlei Liu, Sheng Hu

**Affiliations:** ^1^ Department of Medical Oncology, Tongji Medical College, Hubei Cancer Hospital, Huazhong University of Science and Technology, Wuhan, China; ^2^ College of Life Science and Technology, Huazhong Agricultural University, Wuhan, China; ^3^ National “111” Center for Cellular Regulation and Molecular Pharmaceutics, Key Laboratory of Fermentation Engineering (Ministry of Education), Hubei Provincial Cooperative Innovation Center of Industrial Fermentation, Hubei Key Laboratory of Industrial Microbiology, Hubei University of Technology, Wuhan, Hubei, China; ^4^ Wuhan Binhui Biopharmaceutical Co., Ltd, Wuhan, China; ^5^ Department of Pathology, Tongji Medical College, Hubei Cancer Hospital, Huazhong University of Science and Technology, Wuhan, China

**Keywords:** immunotherapy, oncolytic virus, CAR-T, immunotoxins, dual-specific T cell engagers, FAP, GPC3, synergistic effect

## Abstract

**Background:**

Although the treatment landscape for advanced hepatocellular carcinoma (HCC) has seen significant advancements in the past decade with the introduction of immune checkpoint inhibitors and antiangiogenic drugs, progress has fallen short of expectations. Recently, a novel engineered oncolytic virus (OHSV2) that secretes dual-specific T-cell engagers (DSTEs) targeting the fibroblast activation protein (FAP) was developed and combined with GPC3-targeting CAR-T cells and immunotoxins to exert a synergistic antitumor effect.

**Methods:**

OHSV2-DSTE^FAP5/CD3^ was initially generated by transducing the DSTEs engaging FAP5 on fibroblasts into the backbone of our oncolytic virus OHSV2. An innovative high-order combination was devised in a xenograft mouse model to conceptually explore whether enhanced anti-tumor effects could be achieved. Additionally, the underlying mechanisms of synergistic effects and safety profiles were preliminarily investigated.

**Results:**

OHSV2-DSTE^FAP5/CD3^ effectively targeted and eliminated fibroblasts *in vitro* while maintaining cytotoxicity and inducing immune activation compared to parental OHSV2. *In vivo*, dose-adjusted combination therapy resulted in a remarkable antitumor effect compared to control treatments, leading to tumor regression in 40% of mice without significant toxicity to major organs. Mechanistically, rather than directly depleting fibroblasts, OHSV2-DSTE^FAP5/CD3^ played an essential role in priming T-cell proliferation, infiltration, and activation, and inhibiting the supportive interaction between cancer cells and fibroblasts.

**Conclusions:**

This high-order combination represents a novel multiple-wave immunotherapeutic approach for HCC. Despite being a conceptual exploration, this strategy has demonstrated promising therapeutic efficacy and acceptable safety profiles.

## Graphical Abstract

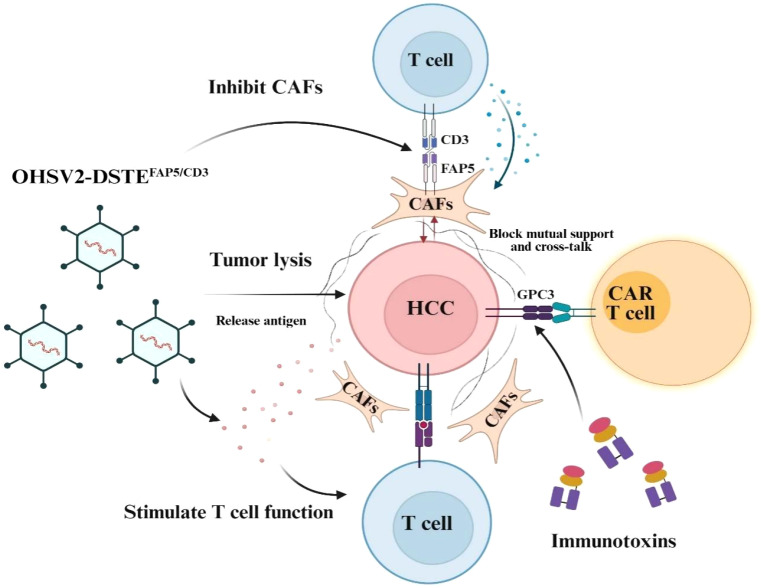

## Background

Hepatocellular carcinoma (HCC) consistently ranks as the third leading cause of cancer-related mortality among all cancers worldwide, highlighting the urgent need for improved therapeutic strategies (1). Over the past decade, the treatment landscape for advanced HCC has undergone significant renovation with the introduction of immune checkpoint inhibitors (ICIs), either as monotherapies, in combination with each other, or plus antiangiogenic drugs. However, therapeutic progress remains suboptimal, as no more than 30% of patients achieve an objective response to the current standard-of-care treatments, with complete responses in fewer than 10% (2, 3). The mechanisms underlying resistance to immunotherapy are exceptionally complex (4), involving intricate interactions among cancer cells, various immune cells, and fibroblasts, which means that anti-cancer battle cannot be won with a single weapon. Nowadays, multiple strategies for combination therapy mechanistically are being explored in parallel, including directly double blocking of a single target on cancer cells, like HER-2, the combination of CAR T cells or ICIs with other drugs facilitating immune cells activation and infiltration or eliminating fibroblasts, such as oncolytic virus or BiTEs (bispecific T-cell engagers). Therefore, the conceptual design of a three-layered combination to overcome resistance and extend clinical response is particularly compelling in the field of HCC treatment, as a high-order combination of multiple immune-based therapies, mainly derived from clinical insights, holds great promise for success both in terms of efficacy and safety (5).

Oncolytic viruses (OVs), as a promising cancer therapeutic approach, not only can selectively replicate within cancer cells and lyse them while sparing normal cells but also induce immunogenic death and subsequently trigger the immune stimulation *in situ* (6), endorsed by definite evidence from Talimogene laherparepvec (T-Vec), a herpes simplex virus type I (HSV-1) approved by the US Food and Drug Administration (FDA) for melanoma in 2015 (7), and our oncolytic herpes simplex virus type II (OHSV2), which has shown efficacy in melanoma and malignant ascites of colon cancer, and other OVs for diversified types of cancer in the preclinical and clinical studies (8–11). However, faced with the fact that the effectiveness of OVs is limited in the vast majority of cancers beyond melanoma, a widely adopted strategy is to fully leverage their function as expression platforms and immune stimulators, using them as a cornerstone for combination therapies, rather than solely focusing on enhancing their direct oncolytic effects.

In the tumor microenvironment (TME), the interaction between cytotoxic T cells (CTLs) and cancer cells resembles that of a predator–prey dynamic within an ecosystem, where predators kill another species (12). Therefore, a logical therapeutic strategy is to directly increase the number of “predators,” such as CAR-T cells. Significant advances have been made in CAR-T cells for hematological malignancies (13, 14), with rapid progress in the context of solid tumors, including our GPC3-targeting CAR-T cells in HCC (15, 16). However, CAR-T cell therapy for solid tumors faces critical challenges, including safety concerns and generally limited antitumor efficacy, albeit with a few promising preclinical outcomes (17). The mechanisms of CAR-T cell resistance are multifaceted, with one major obstacle being the extracellular matrix (ECM) barriers (18) mainly derived from cancer-associated fibroblasts (CAFs), which highly express fibroblast activation protein (FAP) to impede infiltrating of T cells, thereby shaping an immune-suppressive tumor microenvironment, especially in virus-related HCC (19–21).

Accordingly, massive strategies to overcome CAR-T cell resistance and improve safety are currently being developed in preclinical settings (22, 23). Leveraging our extensive experience in OV development, we propose to engineer dual-specific T-cell engagers (DSTEs) into OHSV2, specifically targeting FAP to disrupt CAFs theoretically, which could address CAR-T cells resistance from two dimensions or layers mentioned above, thereby achieving a synergistic anti-tumor effect once combined with GPC3-targeting CAR-T cells for HCC. However, eliminating cancer cells remains a formidable challenge, just like the proverb “cunning rabbit with three holes” (24, 25). Moreover, we have discovered the positive synergy between immunotoxins and CAR-T cells targeting GPC3, consistent with previous findings on the multiple blockades of HER-2 or CD19 (NCT06063317) (26).

In this study, we initially developed OHSV2-DSTE^FAP5/CD3^ and designed an innovative high-order combination by incorporating OHSV2-DSTE^FAP5/CD3^, GPC3-targeting CAR-T cells, and immunotoxins (27) in a mouse HCC model. This approach, distinct from ICIs and anti-angiogenic inhibitors, aims to establish a synergistic process of immune activation and tumor microenvironment remodeling and conceptually explore whether more potent anti-tumor effects can be generated. Additionally, the underlying mechanisms of synergistic effects and safety profiles are preliminarily investigated.

## Methods

### Ethics statement

Peripheral blood was collected from healthy donors following written informed consent, and all animal experiments were conducted according to the institutional review board and research ethics committees of the Huazhong University of Science and Technology, PR China (2019-S1010 and 2019-IEC-S213) to ensure the ethical and welfare of human participants and animals involved in the research process.

### Cell lines

Cell lines in this study, including A375 (human melanoma cell line), A549 (human lung carcinoma cell line), BGC823 (human gastric carcinoma cell line), Hep2 (human head and neck squamous cell carcinoma cell line), HuH-7 (human hepatocellular carcinoma cell line), LoVo (human colon carcinoma cell line), PANC-1 (human pancreatic carcinoma cell line), U87MG (human glioblastoma cell line), 5637 (human bladder carcinoma cell line), MRC-5 (human embryonic lung fibroblasts line), and HEK293 (human embryonic kidney cell line) were obtained from the Cell Bank of Chinese Academy of Sciences (SGST, Shanghai, China). The composition of the culture medium is DME/F12 (Invitrogen, Carlsbad, CA) medium supplemented with 10% fetal bovine serum (HyClone, Logan, UT), 1% L-glutamine (Invitrogen, Carlsbad, CA), and 1% penicillin-streptomycin (Invitrogen, Carlsbad, CA) in a CO_2_ incubator at 37°C for most of these cell lines, while it will undergo appropriate modifications for the other lines. FAP5 cDNA was amplified using FAP-specific primers in 2× qPCR BIO SyGreen Blue Mix Hi-ROX Master Mix (PCR BioSystems) and then transduced into various cancer cells through the well-constructed plasmids to obtain the cancer cells with high expression of FAP5.

### Peripheral blood mononuclear cells, CAR-T cells, and immunotoxins

Peripheral blood mononuclear cells (PBMCs) were isolated by Ficoll separation method (Stem Cell Technologies, Vancouver, BC, Canada). PBMCs were cultured in RPMI 1640 medium supplemented with 200 IU/ml human recombinant interleukin (IL)-2 and activated by Dynabeads™ CD3/CD28 Human T-activator (Cat. 11131D, Thermo Fisher, Waltham, MA) for 3 days according to the manufacturer’s instruction. Alternatively, they can be directly isolated using a blood cell separator (Fresenius CEM TEC., Germany).

The activated PBMCs were directly used in various experiments *in vivo* or *in vitro* or transfected with the lentivirus expressing CARs to obtain CAR-T^HN3^ targeting GPC3 by us (15). In addition, the immunotoxins (scFv fused with PE24, the 24-kDa cytotoxic domain of *Pseudomonas* exotoxin A) targeting the membrane-distal N-lobe of GPC3 previously developed by us, named as J80A-PE24, could suppress tumor growth much greater than naked HN3-PE24 in a xenograft mouse model (27).

### Generation and purification of OHSV2-DSTE^FAP5/CD3^

The DSTE^FAP5/CD3^ was produced by fusing the single-chain variable fragments (scFvs) of anti-FAP 28H1 from patent EP3333194A1-1 and anti-CD3 OKT3 to construct the FAP5/CD3-DSTE expression fragment.

The final fragment VH_FAP5_-VL_FAP5_-VH_CD3_-VL_CD3_ was constructed by Nanjing Kingsley Company and assembled into the pHG52d34.5-CMV shuttle vector (also referred to as pGFP) established in our laboratory for subsequent homologous recombination. The parental virus OHSV2-GFP and OHSV2-DSTE^CD19/CD3^ derived from laboratory were utilized as controls. The genome of OHSV2-DSTE^FAP5/CD3^ was obtained by recombineering and subsequently sequenced by the Tsingke Biotech (Beijing, China). The sequencing results were aligned to the original FAP/CD3-DSTE plasmid sequences by SnapGene software. OHSV2-DSTE^FAP5/CD3^, OHSV2-DSTE^CD19/CD3^, and OHSV2-GFP were used to infect Vero cells for 48–72 h, and the supernatant was collected after the addition of a virus-releasing solution. Afterwards, virus was purified and titrated.

OHSV2-DSTE^FAP5/CD3^ was used to infect Vero cells for 48 h at multiplicity of infection (MOI) of 0.01. After infection, the viral supernatants were collected and purified using High-Affinity Ni-Charged Resin (GenScript). Subsequently, the DSTE^FAP5/CD3^ protein released by infected cells was eluted with the appropriate buffer. The resultant protein solution was purified through 20K Slide-A-Lyzer Dialysis Cassettes according to the manufacturer’s instructions (Thermo Fisher).

### Flow cytometry to analyze cellular components and cytokine levels

Flow cytometry was performed on Accuri C6 cytometer (BD Biosciences, Franklin Lakes, NJ). Human-derived FAP expression was detected with anti-FAP antibody (phycoerythrin, accession number: MIH1, BD Biosciences). T cells were analyzed using the following antibodies: anti-CD3 (APC, accession number: HIT3a, BD Biosciences), anti-CD4 (FITC, accession number: RPA-T4, BD Biosciences), anti-CD8 (FITC, accession number: RPA-T8, BD Biosciences), anti-CD25 (PE, accession number: MA251, BD Biosciences), and anti-CD69 (APC, accession number: FN50, BD Biosciences). Cytokine, including IL2, IL4, IL6, IL10, TNF, and IFN-γ in culture supernatants or peripheral blood, was detected with BD™ Cytometric Bead Array (CBA) Human Th1/Th2 Cytokine Kit II. All flow cytometry data were processed with FlowJo v7.6.5 and FCAP Array v3.0.

### *In vitro* cytotoxicity assays

To investigate the cytotoxicity of free DSTE, oncolytic virus, and T-cell-mediated killing, an average of 1.5 × 10^4^ cancer cells per well was seeded into E-Plate 16 (ACEA Biosciences Inc, San Diego, CA) and co-cultured with pre-activated PBMCs (cancer cells, PBMCs = 1:2) or/and fibroblasts treated by OHSV2-DSTE^FAP5/CD3^ (MOI = 0.1) alone or the other agents and OHSV2 or OHSV2-DSTE^CD19/CD3^ (MOI = 0.1) as control virus. Cellular vitality of cancer cells or others was continuously monitored by the xCELLigence Real-Time Cell Analyzer (ACEA Biosciences Inc, San Diego, CA), following the manufacturer’s protocol. The half-maximal inhibitory concentration (IC50) was calculated by a dose–response inhibition (variable slope) curve with GraphPad Prism V8.0 (GraphPad Software, Inc, La Jolla, CA).

We selected engineering high-expressing FAP5 cancer cells, BGC823-GFP-FAP5 cell lines with three different levels of FAP5 expression to investigate free DSTE or OHSV2-DSTE^FAP5/CD3^ impairment on the cell proliferation *in vitro* continuously in the above-mentioned cell proliferation experiment.

### Reverse transcription quantitative polymerase chain reaction

RNA was extracted from cell lines, mouse tumor tissue, or rabbit spleen, or other components using RNA Simple Total RNA Kit (Tiangen Biotech, Beijing, China), and reverse transcription was carried out to detect levels of IL2RA, GZMB, and PRF1, which are T-cell activation markers by RT-qPCR using 5 × HiScript II QRTSuperMix II (Vazyme Biotech, Nanjing, China) and iTaq Universal SYBR Green Supermix (Bio-Rad, Redmond, WA). Specific primers were designed through the software of Invitrogen’s Vector NTI^®^ Advance 11.5.1. The RT-qPCR procedures briefly were as follows: pre-denaturation at 95°C for 1 min, followed by 40 cycles of 95°C for 5 s, 61°C for 31 s, 95°C for 15 s, and 60°C for 60 s, ending up with heating to 95°C. The relative expression level of the target gene was calculated by the 2^−△△CT^ method with glyceraldehyde 3-phosphate dehydrogenase (GAPDH) gene as an internal reference for three independent biological replicates.

### Animal experiments

6-week-old female BALB/c nude mice were purchased from the Animal Center of Huazhong Agricultural University. Three million HuH-7 cells were subcutaneously injected into the right flank of nude mice feeding under specific pathogen-free conditions. After tumor mass reached the size of approximately 100–200 mm^3^, mice were randomized into seven groups listed in **Table 1** for details. Tumor volume was calculated from the formula: tumor volume (mm^3^) = (length × width × width)/2 by digital calipers. We first carried out dose-found research of OHSV2-DSTE^FAP5/CD3^ *in vivo* in a HuH-7 subcutaneous mouse model intratumorally injected with three different doses of OHSV2-DSTE^FAP5/CD3^ (CCID_50_ = 1 × 10^6^, 1 × 10^7^, and 1 × 10^8^, respectively) or control OHSV2-GFP, once a week, for a total of 4 weeks. Subsequently, formal high-order combination research will be conducted according to the specified schedule and dosages shown in **Table 1**. Mice in the control group and treatment groups were sacrificed for analysis at day 42 after tumor inoculation or at any time due to the oversized tumors.

**Table 1:** The specified schedule of dosages and administration.

Groups^a^ (N=5)	Treatment schedules
Control group	PBS solution 100 µl, intraperitoneal administration once every other day, a total of 4 weeks.
Immunotoxin group	J80A-PE24, administered via the tail vein injection of 3 mg/kg, once a week, for a total of 4 times (27).
Lenvatinib plus anti-PD-1 antibody (pembrolizumab) group	Pembrolizumab, intraperitoneal injection, once every 2 weeks, with a concentration of 2 mg/kg, a total of 2 times. Lenvatinib, 5 mg/kg, was administered by gavage every other day, for a total of 4 weeks.
OHSV2-DSTE^FAP5/CD3^	OHSV2-DSTE^FAP5/CD3^, administered by local injection, once a week, for a total of 4 weeks^b^.
OHSV2-DSTE^FAP5/CD3^ plus J80A-PE24	J80A-PE24 and OHSV2-DSTE^FAP5/CD3^ are administered by the same method above.
OHSV2-DSTE^FAP5/CD3^ plus J80A-PE24 plus anti-PD-1 antibody	J80A-PE24, OHSV2-DSTE^FAP5/CD3^ and anti-PD-1 are administered by the same method above.
OHSV2-DSTE^FAP5/CD3^ plus J80A-PE24 plus CAR-T^HN3^	CAR-T^HN3^ cells, 3 × 10^6^, intravenous injection, once every 2 weeks, a total of 2 times (15, 28). J80A-PE24 and OHSV2-DSTE^FAP5/CD3^ are administered by the same method above.

a PBMCs, 2.5 × 10^6^ cells, were intravenously injected into all groups once every 2 weeks, a total of 2 times.

b The dose of OHSV2-DSTE^FAP5/CD3^ was confirmed by initial dose-finding research.

### Side-effect analysis in mouse tumor model

To examine whether the functions of major organs and systems such as liver, kidney, bone marrow, myocardium, and endocrine system have been impaired by different schedules of administration, we collected peripheral venous blood of mice at the end of the experiment and analyzed levels of various serum enzymes (such as alanine aminotransferase, ALT; aspartate aminotransferase, AST; and creatine kinase, CK), blood glucose, blood lipids, albumin, total bilirubin, creatinine, electrolytes, and cytokine.

### Histopathology and immunohistochemistry analysis in mouse tumor model

Once the mice were sacrificed by cervical dislocation, major organs and partial tumor tissue were collected and immersed in 10% neutral-buffered formalin for fixation. Subsequently, the samples were embedded in paraffin, sectioned (5 μm), and hematoxylin–eosin stained. Additionally, the levels of FAP5 expression were detected by immunohistochemical staining, and grading was assessed by a digital camera (Leica ICC50 HD, Germany) to gather the area and density of the dyed region and calculate the integrated optical density (IOD) value, from five randomly selected fields (Image-Pro Plus 6.0).

These analyses are independently evaluated by two pathologists, and in case of inconsistency, a discussion will be held to make the decision.

### Preliminary analysis of the mechanism by single-cell RNA sequencing

For the high-order combined therapy group, we conducted a preliminary analysis of the mechanism by single-cell RNA sequencing, in addition to T-cell activation characterization and cytokine levels. Tumor tissues were harvested from mice in different treatment groups at the end of the experiments for sample preparation and single-cell RNA sequencing. In short, after digested into cell suspension, they were filtered by a 40-μm strainer and resuspended in the PBS solution to obtain single cells for single-cell sequencing. Single-cell capture was performed by the BD Rhapsody Single-Cell Analysis System (BD Biosciences, Franklin Lakes, NJ) for library construction. Upon preparation of the libraries, they were quantified using the Agilent 2100 Bioanalyzer (Agilent Technologies, Palo Alto, CA) and the Qubit 4.0 (Thermo Fisher Scientific, USA). Finally, the libraries were sequenced on the Illumina NovaSeq 6000 (Illumina, USA), and 300-bp reads (including 150-bp paired-end reads) were generated. Single-cell RNA sequencing data were subjected to multiple analyses including quality control, alignment, clustering, marker gene identification, annotation of clusters, and other analyses.

The clustering results were visualized using t-distributed stochastic neighbor embedding (tSNE) and uniform manifold approximation and projection (UMAP). The marker genes for each cluster were identified using the default parameters through the “FindAllMarkers” function in Seurat. The original clusters were annotated based on the MouseRNAseqData dataset via SingleR (v.1.0.1). T-cell clusters were extracted for further sub-analysis using the subset function. To enhance the distinction between cell types, the ImmGenData dataset was utilized for cluster annotation. Differentially expressed genes (DEGs) related to interleukin (IL) and interferon (IFN) family were screened from the T-cell cluster with |Log2-fold change |  > 0.5 and *p*-value < 0.05 as thresholds and visualized through violin plot by Seurat. Furthermore, genes related to CAFs and T-cell exhaustion were also analyzed.

### Statistical analysis

Quantitative data are usually displayed as mean ± SD from at least three biological replicates and related to the control. Two-tailed unpaired Student’s *t*-tests were performed for comparisons of two independent data sets. Comparisons among three or more groups were performed using two-way ANOVA with Tukey’s multiple comparison test. The statistical significance was denoted as follows: n.s, non-significant; *p < 0.05; **p < 0.01; ***p < 0.001; and ****p < 0.0001. Statistical analysis was conducted by GraphPad Prism 8.0.

## Results

### OHSV2-DSTE^FAP5/CD3^ is successfully constructed

**Figure 1:**
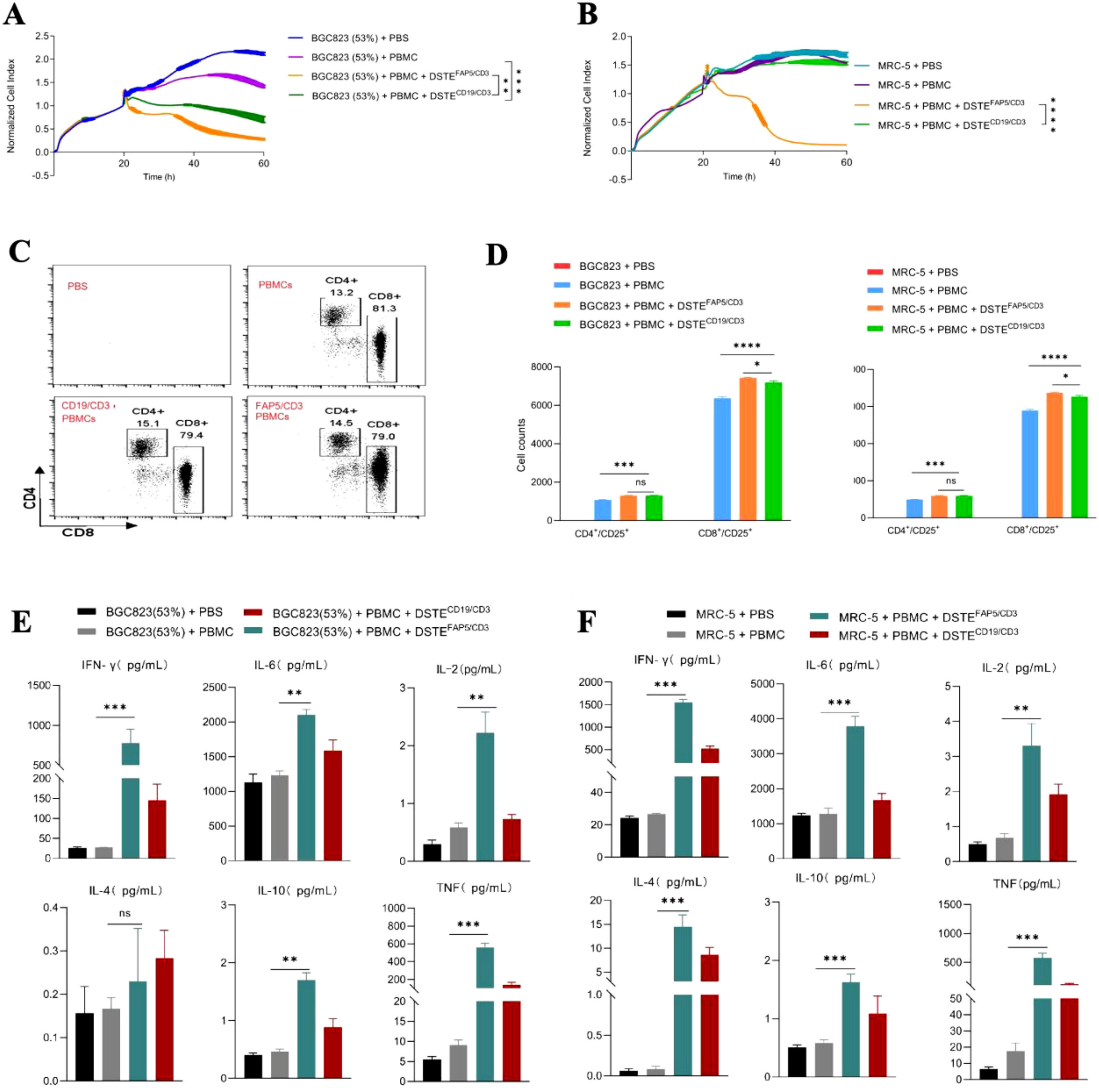
DSTE^FAP5/CD3^ can significantly inhibit the proliferation of fibroblasts or BGC823 cells overexpressing FAP5 with PBMC. **(A)** Tumor cell-killing activity of DSTE^FAP5/CD3^ monotherapy on BGC823-FAP+ cells (53% FAP expression). BGC823 cells were pre-seeded, followed by treatment with oncolytic virus (MOI = 0.1) and PBMCs (cancer cells: PBMC = 1:2). Cell index reflecting cell-killing activity was monitored through xCELLigence Real-Time Cell Analyzer over 60 h (*p < 0.05;** *p* < 0.01; ***p < 0.001; ****p < 0.0001, unpaired two-tailed Student’s t-test.) **(B)** Tumor cell-killing activity of DSTE^FAP5/CD3^ monotherapy on MRC-5 cells. **(C)** Flow cytometric analysis of CD4+ and CD8+ T-cell activation after 60 h co-culture. **(D)** Cell counts of positive CD4 and CD8 T cells following DSTE^FAP5/CD3^ treatment. **(E, F)** Cytokine secretion profiles (IFN-γ, IL-2, IL-4, IL-6, IL-10, and TNF) in supernatants from **(E)** BGC823 and **(F)** MRC-5 co-cultures with DSTE^FAP5/CD3^, measured by BD™ Cytometric Bead Array (CBA) Human Th1/Th2 Cytokine Kit II. Data are represented as mean ± SD (n = 3 biological replicates; unpaired two-tailed Student’s t-test).

DSTE^FAP5/CD3^ was constructed using the FAP5 monoclonal antibody 28H1, paired with the CD3 monoclonal antibody OKT3. In combination with peripheral blood mononuclear cells (PBMCs), DSTE^FAP5/CD3^ significantly inhibited the proliferation of BGC823 cells overexpressing FAP5, and fibroblasts, in a time-dependent manner, with maximal effect at 60 h (95% tumor cell apoptosis) monitored by CellInsight CX5 high-content system (**Figures 1A, B**), compared with DSTE^CD19/CD3^ (preserved in our laboratory only utilized as a control).

Mechanistically, our results showed a significant upregulation in the proliferation of both CD4+ and CD8+ T cells in the DSTEFAP5/CD3 plus PBMC group (2,557 ± 76.59 and 9,362 ± 101.8, respectively), compared to the control group (1,141 ± 79.23 and 6,215 ± 120.7, respectively, *p* < 0.05) (**Figures 1C, D**). In addition, the proliferation amplitude between CD4+ and CD8+ T cells was approximately similar in DSTE^FAP5/CD3^ plus PBMC group. Furthermore, multiple cytokine levels in the supernatant were found to be significantly elevated, particularly IFN-γ, IL-6, and TNF (**Figures 1E, F**).

**Figure 2:**
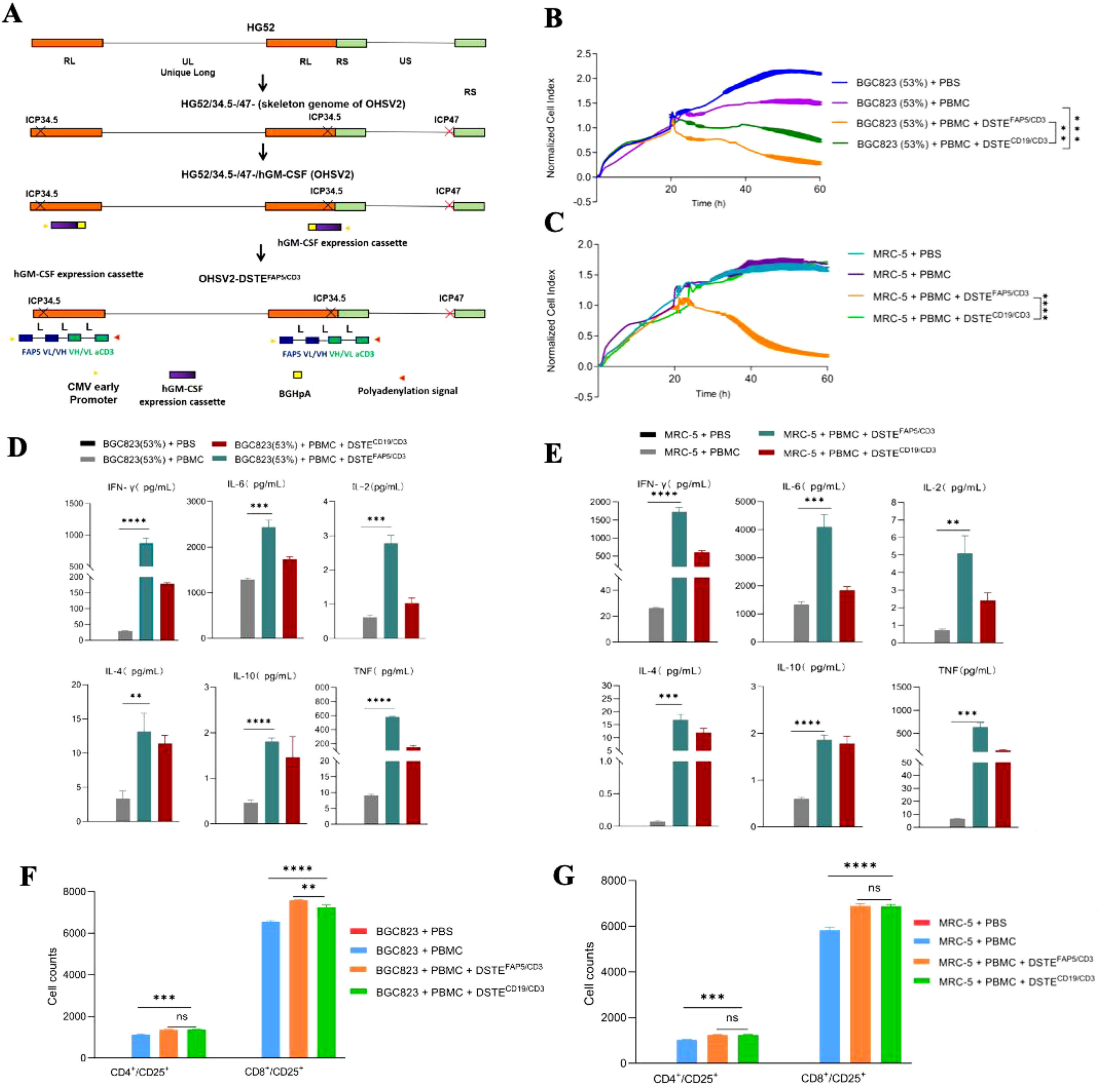
Construction and functional analysis of OHSV2-DSTE^FAP5/CD3^. **(A)** Schematic representation of OHSV2-DSTE^FAP5/CD3^ construction. HG52, herpes simplex virus type 2 virus strain; ICP34.5/ICP47, infected cell protein 34.5/47; RL, repeat long region; RS, repeat short region; UL/US, unique long/short; GFP, enhanced green fluorescent protein; CMV, cytomegalovirus. **(B, C)** DSTE^FAP5/CD3^ secreted from OHSV2 inhibited the proliferation of BGC 823 cells overexpressing FAP5 (53%) and MRC-5 cells co-cultured with PBMC through activating T cells *in vitro* (unpaired two-tailed Student’s t-test, **p < 0.01; ***p < 0.001; ****p < 0.0001). **(D, E)** DSTE^FAP5/CD3^ secreted from OHSV2 induced cytokines production, including IL-2, IL-4, IL-6, IL-10, TNF, and IFN-γin BGC 823 cells overexpressing FAP5 and MRC-5 cells *in vitro* by BD™ Cytometric Bead Array (CBA) Human Th1/Th2 Cytokine Kit II. Data are presented as means ± SD (n=3 biological replicates; unpaired and two-tailed Student’s t-test.). **(F, G)** Cell counts of CD4+ and CD8+ T cells on BGC 823 cells overexpressing FAP5 and MRC-5 cells after incubation with PBMC, assessed by flow cytometry. ns, non-significant.

Next, DSTE^FAP5/CD3^ was transduced into OHSV2 to generate a novel virus, named OHSV2-DSTE^FAP5/CD3^ (**Figure 2A**), which was used to infect Vero cells. At 60 h post-infection, the concentration of DSTE^FAP5/CD3^ reached 0.12 ng/ml, despite lower than that achieved by recombination methods, indicating that insertion of DSTE gene fragments did not impair replication of OHSV2 relative. Subsequently, we collected the supernatant and found that DSTE^FAP5/CD3^ also exhibited killing activity (**Figures 2B, C**). Moreover, the levels of DSTE^FAP5/CD3^-induced cytokines, including IL-2, IL-4, IL-6, IL-10, TNF, and IFN-γ were markedly elevated *in vitro*, indicating the activation of T cells and immune function enhancement in BGC823 cells overexpressing FAP5 cells and MRC-5 cells (**Figures 2D–G**), suggesting that OHSV2-DSTE^FAP5/CD3^ is successfully constructed; thus, in-depth exploration is necessary to achieve our therapeutic objectives.

### OHSV2-DSTE^FAP5/CD3^ exhibits killing activity and simultaneously stimulates PBMCs *in vitro*

**Figure 3:**
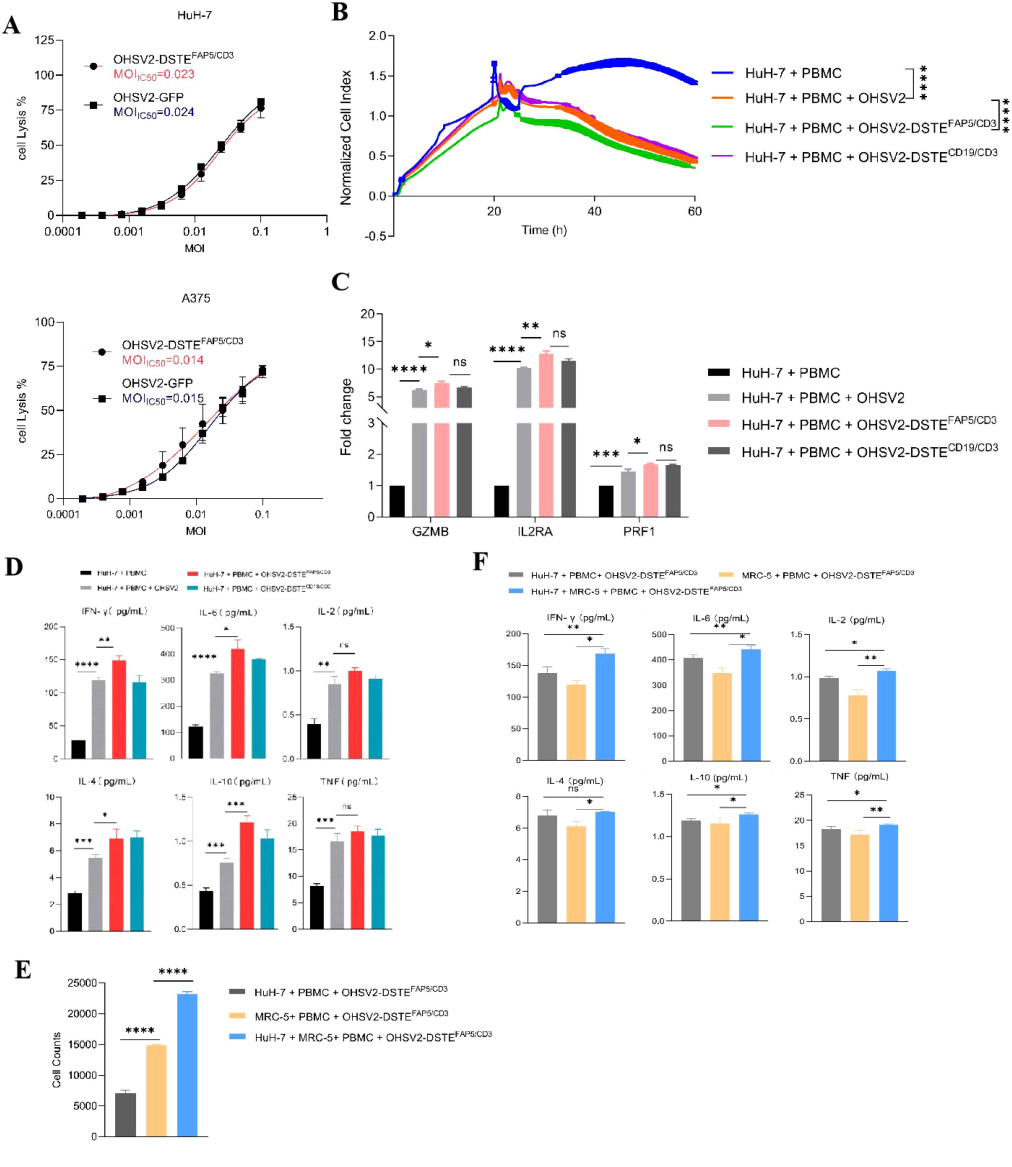
Anti-tumor efficacies of OHSV2-DSTE^FAP5/CD3^ *in vitro*. **(A)** Oncolytic activities of OHSV2-DSTE^FAP5/CD3^ in HuH-7 and A375 cell lines. HuH-7 and A375 cell lines were incubated with serial dilutions of OHSV2-GFP or OHSV2-DSTE^FAP5/CD3^ by MTT assay. On day 3 post-infection, cell viability was detected, and the IC50 was calculated for each virus (n=4 biological replicates per experiment). MOI, multiplicity of infection; IC50, half-maximal inhibitory concentration. **(B)** Tumor cell-killing activity of OHSV2-DSTE^FAP5/CD3^ monotherapy in Huh-7 cells. HuH-7 cells were seeded for 24 h before the experiment. Afterwards, oncolytic virus (MOI=0.1) and PBMCs (cancel cells: PBMCs =1: 2) were added and incubated. The cell index reflecting cell-killing activity was monitored through xCELLigence Real-Time Cell Analyzer over 60 h Unpaired two-tailed Student’s t-test. ****p<0.0001. **(F)** Cytokine levels, including IL-2, IL-4, IL-6, IL-10, TNF, and IFN-γ, significantly increased in the co-culture group containing both cancer cells and fibroblasts compared to groups with either cancer cells or fibroblasts alone. **(C)** Expressions of T-cell activation-related genes, including granzyme B (GZMB), interleukin-2 receptor A (IL2RA), and Perforin 1 (PRF1) through RT qPCR. RNA was extracted from PBMCs, reverse-transcribed into cDNA, and analyzed. Data are presented as mean ± SD (n = 3 biological replicates; unpaired Student’s two-tailed t-test; ns, not significant; *p<0.05; **p<0.01; ***p<0.001; ****p<0.0001). **(D)** Levels of cytokines IL2, IL4, IL6, IL10, TNF, and IFN-γ induced by OHSV2-DSTE^FAP5/CD3^- *in vitro*, measured using BD™ Cytometric Bead Array (CBA) Human Th1/Th2 Cytokine Kit II. **(E)** Cell counts significantly increased when Huh-7 cells were co-cultured with MRC-5 in OHSV2-DSTE^FAP5/CD3^ treatment, compared to Huh-7 and MRC-5 cell alone groups. **(F)** The levels of cytokines, including IL-2, IL-4, IL-6, IL-10, TNF, and IFN-γ, significantly increased in the group co-cultured with both cancer cells and fibroblasts compared to groups with either cancer cells or fibroblasts alone.

To further explore the lytic function of OHSV2-DSTE^FAP5/CD3^, we initially examined human melanoma cells, which demonstrate relatively higher sensitivity to most immunotherapy drugs. Our findings showed that OHSV2-DSTE^FAP5/CD3^ can exhibit uncompromising lytic activity compared to its parental virus (**Figures 3A, B**). Furthermore, the *in vitro* anti-tumor efficacy of OHSV2-DSTE^FAP5/CD3^ against HuH-7 cells was significantly greater than that observed in CT-26, MC-38, 4T1, and BGC823 cell lines lacking FAP5 expression, and analogous to control virus, such as OHSV2-DSTE^CD19/CD3^ or OHSV2, albeit weaker than its effect on melanoma cells (**Table 2**). These results imply that HuH-7 cells possess an inherent susceptibility to immune-mediated killing induced by OHSV2-DSTE^FAP5/CD3^, accompanied by significant T-cell activation (**Figures 3C, D**), prompting our team to select this cell line for further investigation.

**Table 2:** The functional parameters of OHSV2-DSTE^FAP5/CD3^ *in vitro* at 48 h (MOI = 0.2).

Cell lines	Cytopathic effects (%)	hGM-CSF (ng/ml)	DSTE^FAP5/CD3^ (ng/ml)
HT-29	81.5	105.2	81.1
HuH-7	89.6	113.3	86.4
BGC823	82.2	108.9	78.7
A375	99.9	152.1	102.2
A549	83.5	101.5	77.8

OHSV2 demonstrates limited replication capacity and consequently fails to directly eliminate fibroblasts or other nonepithelial stromal cells with normal antiviral pathways, such as interferon secretion. Furthermore, co-culturing cancer cells and fibroblasts may affect their respective survival rates, although these effects can vary depending on multiple factors. Hence, co-cultivation of HuH-7 and FAP-expressing stromal fibroblasts was lastly carried out. The results suggested that OHSV2-DSTE^FAP5/CD3^ exhibited direct HuH-7 cell killing and T-cell-mediated fibroblast elimination (**Figure 3E**). However, the potential enhancement of oncolytic effects through CD3-induced T-cell clustering activation remains to be elucidated. Moreover, OHSV2-DSTE^FAP5/CD3^ exhibits negligible cytotoxicity toward fibroblasts in the absence of cancer cells or PBMCs, indicating that its function is constrained by environmental conditions, which may confer potential safety advantages for further exploration and even clinical development.

Furthermore, the levels of multiple cytokines, including IL-2, IL-4, IL-6, IL-10, TNF, and IFN-γ in the supernatant of the OHSV2-DSTE^FAP5/CD3^ group co-cultured with cancer cells and fibroblasts, showed a significant increase compared to groups with either cell type alone (**Figure 3F**). This suggests that T cells derived from PBMCs are primarily activated by OHSV2, with DSTE playing a secondary regulatory role. It should be noted that our interpretation is based solely on a correlation study and requires further validation.

**Figure 4:**
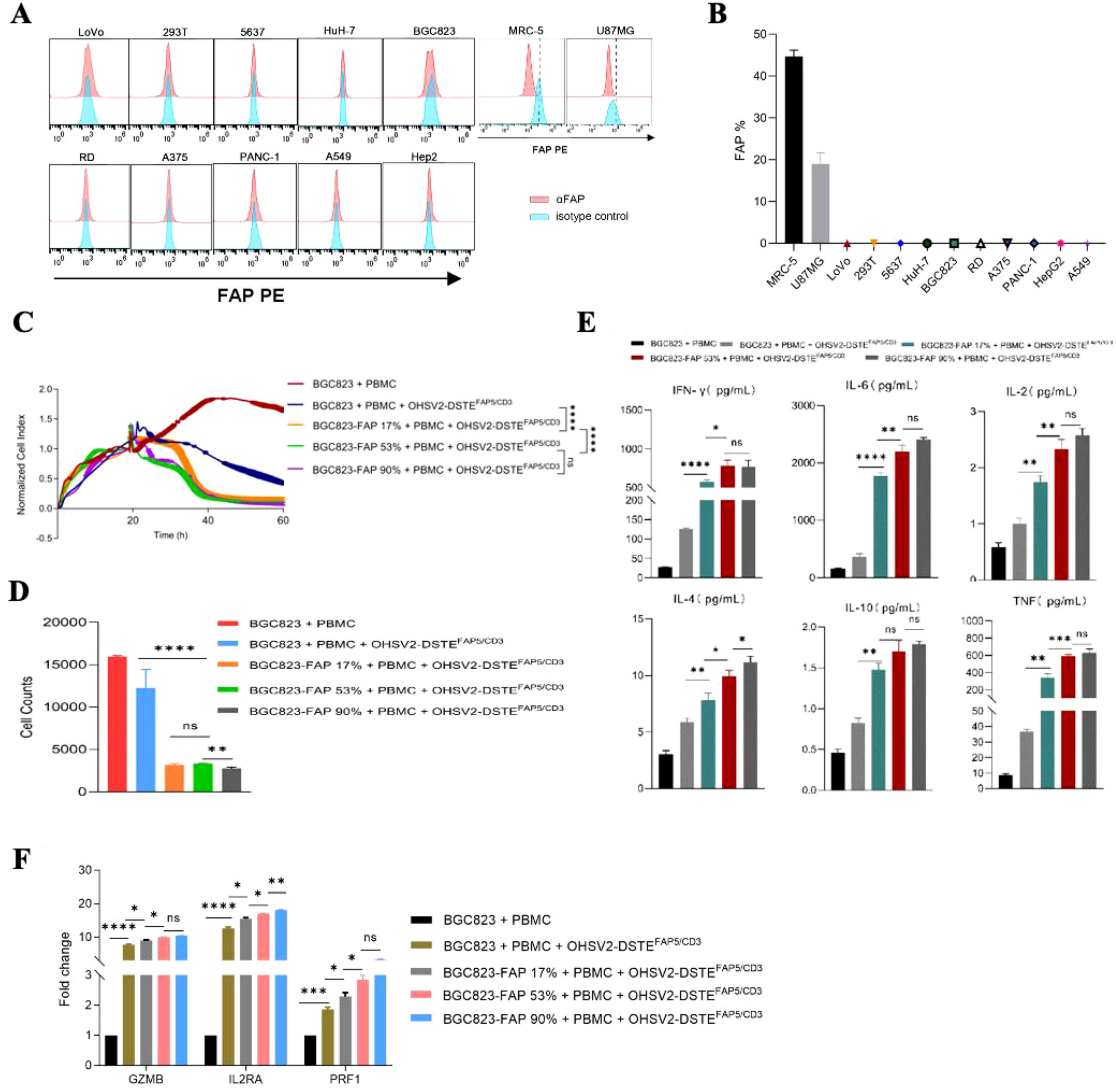
Anti-tumor efficacy of OHSV2-DSTE^FAP5/CD3^ and its effects on T cells in high-FAP5 setting *in vitro*. **(A)** Expression of FAP in different cancer cell lines. Cancer cells were collected, stained with anti-FAP antibody, and analyzed by flow cytometry. **(B)** FAP positivity rate in different tumor cell lines based on the FAP expression analysis in part **(A)**. **(C)** Tumor cell-killing activity of OHSV2-DSTE^FAP5/CD3^ in BGC823-FAP+ cells with different FAP expression levels. Tumor cells were seeded before the experiment. Afterwards, oncolytic virus (MOI=0.1) and PBMCs (cancer cells: PBMCs =1: 2) were added and incubated. Cell index reflecting cell-killing activity was monitored through xCELLigence Real-Time Cell Analyzer over 60 h Unpaired two-tailed Student’s t-test. ns, not significant; *p<0.05; **p<0.01; ***p<0.001; ****p<0.0001. **(D)** Cell counts of tumor cells in OV treatment groups following OHSV2-DSTE^FAP5/CD3^ infection. Data are presented as mean ± SD. (n = 3 biological replicates; unpaired Student’s two-tailed t-test). **(E)** Levels of cytokines including IFN-γ, IL-6, IL-2, IL-4, IL-10, and TNF induced by OHSV2-DSTE^FAP5/CD3^ in BGC823-FAP+ cells with different FAP expression levels. **(F)** Expressions of T-cell activation-related genes, including GZMB, IL2RA, and PRF1, as determined by RT-qPCR. RNA was extracted from PBMCs, reverse transcribed into cDNA, and analyzed.

Subsequently, the FAP5-targeted function of OHSV2-DSTE^FAP5/CD3^ in high FAP5 expression setting was explored. Initial assessment of FAP5 expression levels across various tumor cell lines revealed that only MRC-5 and U87MG cell lines exhibited high expression levels of FAP5, with an expression of 45% and 18%, respectively (**Figures 4A, B**). Conversely, FAP5 expression was nearly undetectable in other malignant tumor cells such as A375 and A549, consistent with its established role as a marker for tumor stroma. Following 48-h co-incubation, OHSV2-DSTE^FAP5/CD3^ can perform greater cancer cell lysis in a FAP5 level-dependent manner, through comparing three cell lines of BGC823 with 90%, 53%, and 17% of FAP5 expression levels to parental BGC823 (**Figures 4C, D**) and HuH-7 (*p* < 0.05), suggesting that the additive antitumor activity of OHSV2-DSTE^FAP5/CD3^ is dependent on the direct on-target effect of DSTE^FAP5/CD3^. Moreover, T cells were significantly activated by OHSV2-DSTE^FAP5/CD3^, supported by the upregulation of T-cell activation-related genes, including GZMB, IL-2RA, and PRF1, and increased secretion of cytokines (IL2, IL4, IL6, IL10, TNF, and IFN-γ) (**Figures 4E, F**).

These results collectively demonstrated that OHSV2 is an outstanding platform to express DSTE^FAP5/CD3^, and HCC is a preferred prey for OHSV2-DSTE^FAP5/CD3^ because of at least triple effects of promoting proliferation and activation of T cells, along with a direct oncolytic effect observed *in vitro*.

### Screening for appropriate combination regimens based on OHSV2-DSTE^FAP5/CD3^ *in vitro*

Based on the aforementioned findings, OHSV2-DSTE^FAP5/CD3^ has been characterized as possessing robust oncolytic activity, moderate stromal elimination capability, significant immune activation potential, and a favorable safety profile. In addition, our previous research demonstrated that the CAR-T cells (CAR-T^HN3^) and immunotoxins (J80A-PE24) targeting GPC3 developed in our laboratory exhibited significantly greater cytotoxicity than single-agent treatments, although further optimization is warranted. Therefore, we propose a high-order combination regimen comprising OHSV2-DSTE^FAP5/CD3^, CAR-T^HN3^, and J80A-PE24. This combination is anticipated to exhibit synergistic anti-tumor effects and is theoretically feasible while circumventing challenges associated with integrating therapeutics from different teams in future clinical studies.

**Figure 5:**
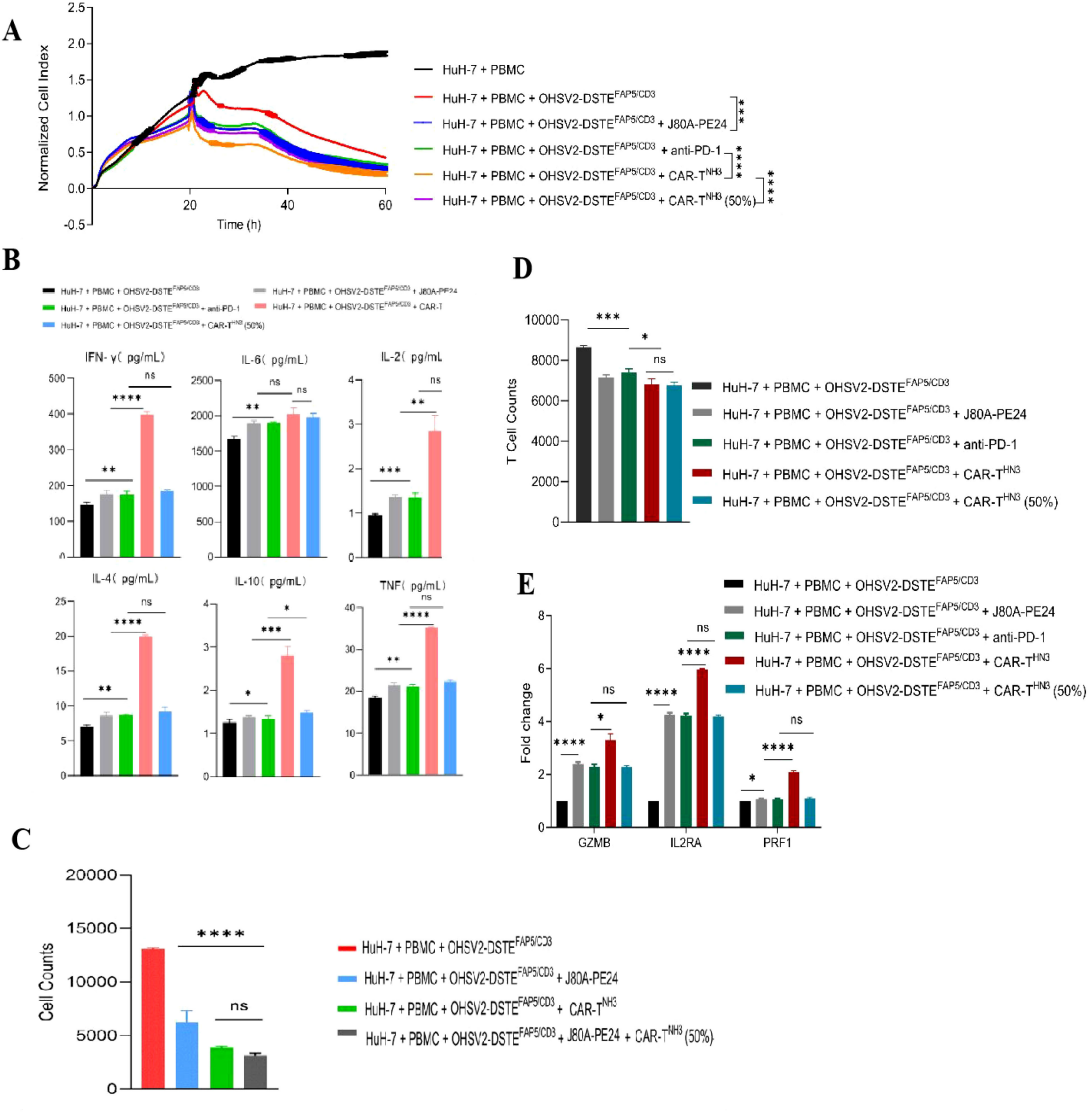
Screening for appropriate combination regimens based on OHSV2-DSTE^FAP5/CD3^ *in vitro*. **(A)** Tumor cell-killing activity of combination therapy of OHSV2-DSTE^FAP5/CD3^ ± immunotoxins ± PD-1 monoclonal antibody in HuH-7 cells through xCELLigence Real-Time Cell Analyzer. Unpaired two-tailed Student’s t-test. ***p<0.001; ****p<0.0001. **(B)** Levels of the combination cytokines including IFN-γ, IL-6, IL-2, IL-4, IL-10, and TNF induced OHSV2-DSTE^FAP5/CD3^ in HuH-7 cells. Data are presented as mean ± SD (n = 3 biological replicates; unpaired Student’s two-tailed t-test; ns, not significant; *p<0.05; **p<0.01; ***p<0.001; ****p<0.0001). **(C)** Tumor cell counts in OHSV2-DSTE^FAP5/CD3^ combination treatment groups. **(D)** T-cell numbers in OHSV2-DSTE^FAP5/CD3^ combination treatment groups. **(E)** Expressions of T-cell activation-related genes, including GZMB, IL2RA, and PRF1 through RT qPCR.

First, we found that the combination of OHSV2-DSTE^FAP5/CD3^ with GPC3-targeting immunotoxins exhibited significantly enhanced cytotoxicity against HuH-7 cells (*p*<0.05) compared to monotherapies (**Figure 5A**). It is worth mentioning that OHSV2-DSTE^FAP5/CD3^ plus CAR-T^HN3^ induced higher cytokine levels than single-agent treatment (**Figure 5B**), which is a major contributor to severe toxicity in CAR-T cell therapy, although showing significant synergistic anti-tumor effects. Thus, we reduced the CAR-T cells dosage by 50% and found that cytokine levels were similar to those in the other treatments but with a 30% decrease in efficacy. Therefore, this adjusted dosing strategy was employed in subsequent *in vivo* experiments to balance safety and efficacy. Lastly, what exceeded our expectations is that adding GPC3-targeting immunotoxins into OHSV2-DSTE^FAP5/CD3^ and GPC3-targeting CAR T cells group resulted in a significant increase in the killing effect on tumor cells (**Figure 5C**). We speculated that this may be due to a saturation of cytotoxic potential for the latter combination *in vitro*, where no major barriers hinder effector cell infiltration and activation. However, this scenario is difficult to replicate *in vivo*, especially in solid tumors. Therefore, this strategy remains valuable to be explored in HuH-7 mice models.

In addition, our results showed that T-cell activation markers, including GZMB, IL2RA, and PRF1, were more significantly upregulated in the OHSV2-DSTE^FAP5/CD3^ plus CAR-T cell group compared to the OHSV2-DSTE^FAP5/CD3^ or CAR-T cell group, suggesting an enhancement in T-cell effector function, consistent with the findings from plenty of preclinical studies (**Figures 5D, E**).

### OHSV2-DSTE^FAP5/CD3^ mediates tumor lysis *in vivo*

Given that GPC3-targeting immunotoxins and GPC3-targeting CAR T cells have already been explored *in vivo* in our previous research, we conducted a dose-finding research of OHSV2-DSTE^FAP5/CD3^ in HuH-7 subcutaneous mouse model, intratumorally injected with three different doses of OHSV2-DSTE^FAP5/CD3^ (CCID_50_ = 1×10^6^, 1×10^7^, and 1×10^8^) or control OHSV2-GFP. A relatively low dose of 1×10^7^ was selected for subsequent experiments, as it exhibited more significant tumor regression than the 1×10^6^ group or OHSV2-GFP group (**Supplementary Figure 1**) while showing comparable efficacy to the 1×10^8^ group. However, a tendency of gradual acceleration in tumor growth was observed at a later stage, even though OHSV2-DSTE^FAP5/CD3^ treatment effectively inhibited the tumor growth initially. These observations suggested that resistance to oncolytic viruses necessitates overcoming through the development of combination therapies.

**Figure 6:**
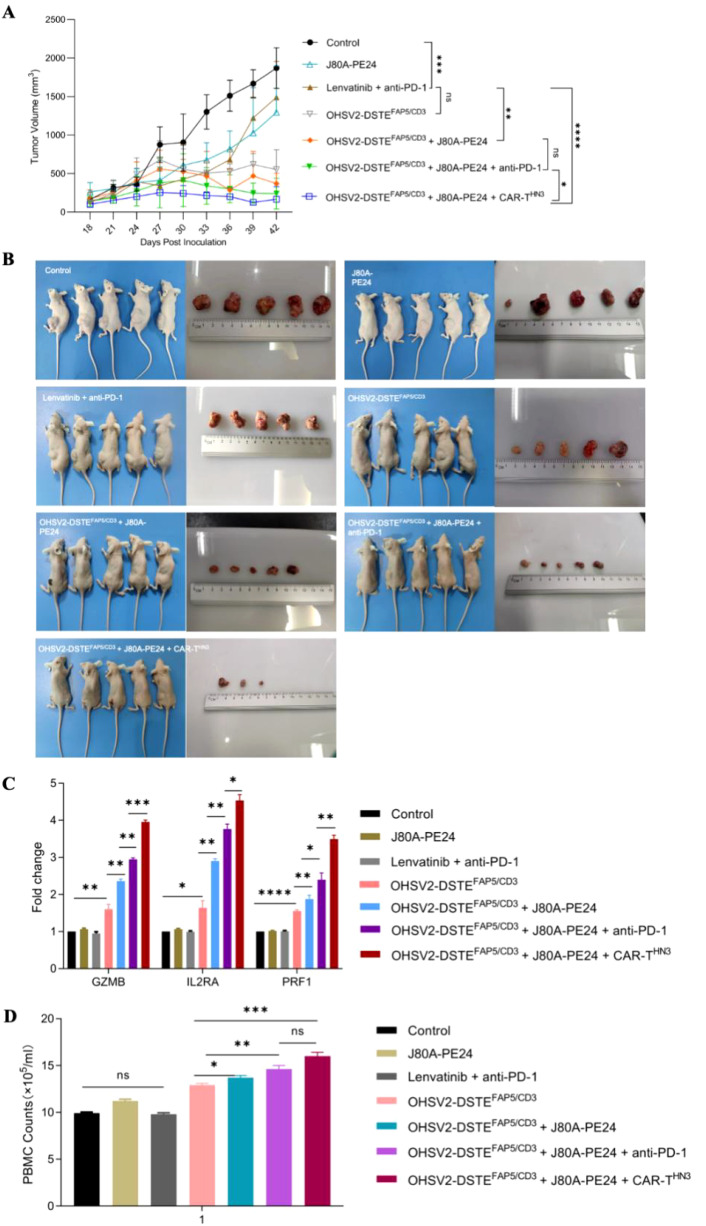
OHSV2-DSTE^FAP5/CD3^-based combined therapy inhibits the tumor growth of HuH-7 *in vivo*. **(A)** Tumor growth curves of six different treatment groups and one control group. The control group consisted of untreated mice, while the treatment groups included immunotoxins J80A-PE24 + PBMCs, Lenvatinib + pembrolizumab (anti-PD-1) + PBMCs, OHSV2-DSTE^FAP5/CD3^ + PBMCs, OHSV2-DSTE^FAP5/CD3^ + J80A-PE24 + PBMCs, and OHSV2-DSTE^FAP5/CD3^ + J80A-PE24 + pembrolizumab + PBMCs, OHSV2-DSTE^FAP5/CD3^ + J80A-PE24 + CAR-T^HN3^ + PBMCs. The tumor volumes of mice were measured every 3 days. All mice were euthanized by cervical dislocation on day 42. Data were presented as mean ± SD and analyzed using an unpaired two-tailed Student’s t-test. ns, not significant; *p < 0.05; **p<0.01; ***p<0.001; ****p<0.0001. **(B)** Images of mice in the control group and the six treatment groups and the corresponding tumor from each mouse (n=5 mice per group). **(C)** Expressions of T-cell activation-related genes, including GZMB, IL2RA, and PRF1. Data are presented as mean ± SD from three biological replicates and analyzed using an unpaired two-tailed Student’s *t*-test. **(D)** PBMCs counts isolated from mice with OHSV2-DSTE^FAP5/CD3^-based combined therapy.

Subsequently, we found that the innovative three-order combination by incorporating OHSV2-DSTE^FAP5/CD3^, GPC3-targeting CAR-T cells (CAR-T^HN3^), and immunotoxins (J80A-PE24) exerted the most potent anti-tumor effect in the mouse HCC model, as evidenced by the smallest tumor volumes at day 42 (**Figures 6A, B**). Unexpectedly, complete tumor eradication was observed in 40% of the mice (two out of five). Poor tumor growth could be ruled out, as the tumor volume increased to approximately 400 mm^3^ during the initial stage of treatment. Moreover, all other mice exhibited normal tumor growth kinetics before receiving this triple-agent combination therapy. In addition, hallmark of T-cell activation and PBMC counts were more pronounced in OHSV2-DSTE^FAP5/CD3^-based combined therapy group (**Figures 6C, D**). Therefore, our conceptual design of a high-order combination therapy integrating OHSV2-DSTE^FAP5/CD3^, CAR-T cells, and immunotoxins—three agents with distinct, non-overlapping mechanisms—was successfully transformed into reality. Our results also demonstrated that OHSV2-DSTE^FAP5/CD3^-based treatment was superior to anti-PD-1 antibody-based treatment, although this was not a head-to-head comparison between single agents. Furthermore, the OHSV2-DSTE^FAP5/CD3^-based combinations were more effective than single- or dual-agent regimens, including lenvatinib plus anti-PD-1 treatment, which has shown high tumor response rates in clinical trials but has not been approved by the FDA recently.

### OHSV2-DSTE^FAP5/CD3^ exhibits a good safety profile *in vivo*

To evaluate the safety of OHSV2-DSTE^FAP5/CD3^ in combination with GPC3-targeting CAR-T cells (CAR-T^HN3^) and immunotoxins (J80A-PE24), a series of tests were conducted. Given the potential for increased toxicity due to the combined effects of these agents and the known risks of severe adverse events associated with CAR-T cell therapy (e.g., cytokine release syndrome or neurotoxicity), we selected a moderate dose of OVs and a half-dose of CAR-T cells.

**Figure 7:**
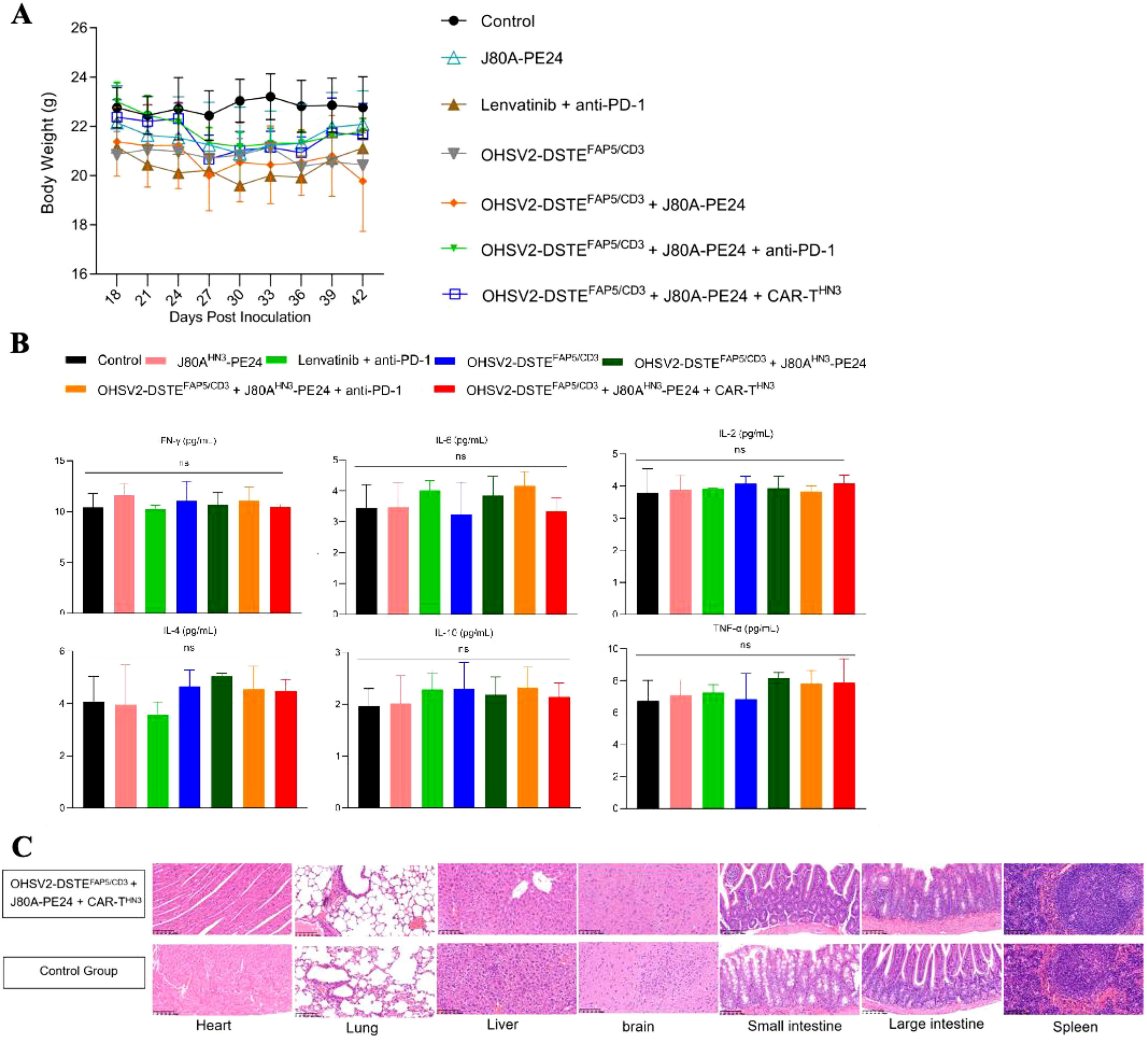
Safety evaluation of OHSV2-DSTE^FAP5/CD3^. **(A)** Body weights of HuH-7 tumor-bearing mice in all groups from day 18 to day 42 post-inoculation. **(B)** Levels of cytokines IFN-γ, IL-6, IL-2, IL-4, IL-10, and TNF *in vivo*, measured using BD™ Cytometric Bead Array (CBA) Human Th1/Th2 Cytokine Kit II. Blood samples were collected from the retro-orbital sinus of mice in all the groups. Data are presented as mean ± SD (n=5 mice per group, two-way ANOVA with Tukey’s multiple comparisons test). **(C)** Histopathological assays of the heart, lung, liver, brain, small intestine, large intestine, and spleen of HuH-7 tumor-bearing mice from OHSV2-DSTE^FAP5/CD3^-based triple regimen groups (OHSV2-DSTE^FAP5/CD3^ + J80A-PE24 + CAR-T^HN3^), evaluated by hematoxylin and eosin (H&E) (200×).

First, mouse body weights showed no significant descending or differences across different groups through regular monitoring (**Figure 7A**). At the same time, the parameters reflecting organ function, including the glutathione transaminases, phosphocreatine kinases, myocardial enzymes, albumin, bilirubin, creatinine, hemocyte numbers, and glucose levels in peripheral blood, occasionally displayed, at most, grade 1 deterioration (**Supplementary Table S1**), suggesting that all treatment schemes, especially our novel triple-agent combination, did not induce overlying toxic effects in major organs or tissues, such as the liver, kidney, myocardium, or bone marrow. Furthermore, these findings revealed a decoupling between therapeutic efficacy and toxicity, although the underlying mechanisms remain to be elucidated at present. Moreover, serum cytokine levels were not significantly increased in the triple-combination group at the end of the experiment on day 42 compared to controls (**Figure 7B**), suggesting that although the treatments activated immune responses within the tumor microenvironment, they did not lead to excessive systemic cytokine release. Additionally, histopathological analysis revealed no significant tissue damage in the monotherapy group (OHSV2-DSTE^FAP5/CD3^) (**Figure 7C**) or in combination therapy groups compared to the blank control.

Taken together, these findings have largely dispelled our initial concerns regarding the potential for severe on-tumor and off-target side effects, such as cytokine storms, interstitial pneumonia, and hepatic impairment, resulting from combination therapy containing OHSV2-DSTE^FAP5/CD3^, because of the relatively low specificity of FAP and GPC3 target antigens, of course, suggesting that this novel strategy integrating different immune therapeutic drugs is worth further clinical development.

### A preliminary exploration of the mechanisms underlying the novel strategy integrating OHSV2-DSTE^FAP5/CD3^ with other immune therapeutic agents

Further exploration of the mechanism underlying the novel strategy combining OHSV2-DSTE^FAP5/CD3^ with other immune therapeutic drugs is still necessary. Although not tremendously critical for clinical translation efforts, such insights can guide the iterative development of next-generation therapeutics. To this end, various cell populations, probably including CD3+ T cells, CAFs, and other immune cells T cells, were isolated from tumor tissues across different treatment groups and identified seven distinct cell clusters (types) based on gene marker expression profiles by single-cell sequencing (**Supplementary Figure S2**).

**Figure 8:**
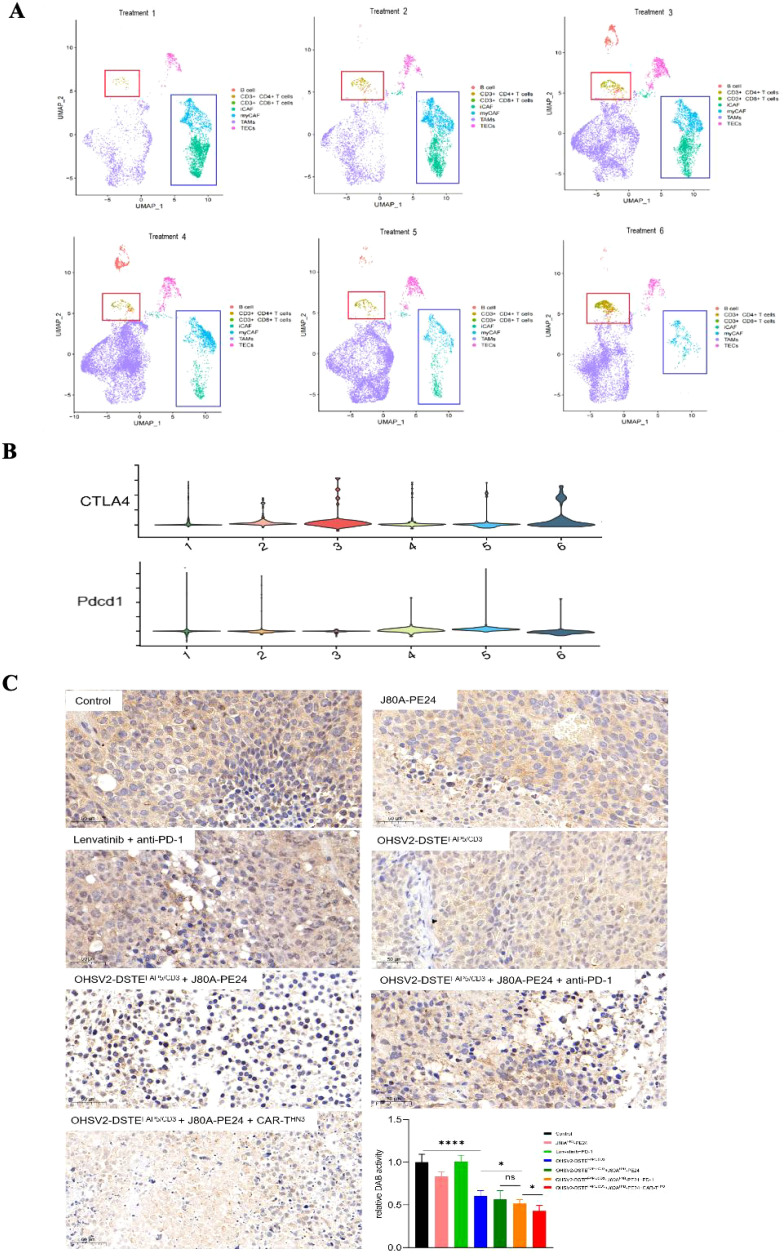
Narrow-scoped mechanism analysis on OHSV2-DSTE^FAP5/CD3^-based triple-agent therapy. **(A)** UMPA plot of different lymphocyte subsets and CAFs derived from tumor tissues of each treatment group. The expressions of activation genes in CD3+ T cells was analyzed in the following OHSV2-DSTE^FAP5/CD3^-based therapy groups: treatment 1, immunotoxins J80A-PE24; treatment 2, Lenvatinib + pembrolizumab (anti-PD-1); treatment 3, OHSV2-DSTE^FAP5/CD3^; treatment 4, OHSV2-DSTE^FAP5/CD3^ + J80A-PE24; treatment 5, OHSV2-DSTE^FAP5/CD3^ + J80A-PE24 + pembrolizumab; and treatment 6, OHSV2-DSTE^FAP5/CD3^ + J80A-PE24 + CAR-T^HN3^). **(B)** Violin plot of expressions of inflammatory CAFs (iCAF) and myofibroblastic *CAFs* (myCAF) on their respective marker genes. **(C)** Immunohistochemical analysis of FAP expression in CAFs from tumor tissues of all seven groups (200×). FAP expression was detected across all the OHSV2-DSTE^FAP5/CD3^-based therapy groups. Scale bar = 5 μm. Blue, nuclear; brown, FAP. *p< 0.05 and ****p < 0.0001.

Notably, the proportions of CD4+ and CD8+ T cells were significantly increased in the OHSV2-DSTE^FAP5/CD3^-based triple-agent regimen groups (including OHSV2-DSTE^FAP5/CD3^ + J80A-PE24 + CAR-T^HN3^ group and OHSV2-DSTE^FAP5/CD3^ + J80A-PE24 + anti-PD-1 group), compared to other treatment groups (**Supplementary Table S2**), however, there is no significant difference between two triple-agent regimen groups or among other dual-agent regimen groups, besides blank control (**Figures 8A, B**). The gene amplification related to CD8+ T cell exhaustion was also observed, suggesting that our constructed OHSV2-DSTE^FAP5/CD3^ effectively activates T cells *in vivo*.

Lastly, following the expression profiling of CAFs, we categorized them into myofibroblast CAFs (myCAFs) and inflammatory CAFs (iCAFs). Remarkably, the OHSV2-DSTE^FAP5/CD3^ + J80A-PE24 + CAR-T^HN3^ group demonstrated the most pronounced CAF depletion among all treatment groups (**Figure 8A**). Interestingly, the gene expression patterns of the OHSV2-DSTE^FAP5/CD3^ +J80A-PE24+CAR-T^HN3^ group did not significantly differ from those of other treatment groups (**Supplementary Figure S2**), suggesting that distinct treatment modalities did not impact the overall gene expression profiles of CAFs. To further corroborate the depletion of CAFs in the tumor microenvironment, we conducted immunohistochemistry to evaluate the expression of FAPs in the OHSV2-DSTE^FAP5/CD3^ + CAR-T cells + immunotoxin combination group and revealed a significant reduction in CAFs, contrasting with the effect observed in the OHSV2-DSTE^FAP5/CD3^ monotherapy group (**Figure 8C**), where the impact on CAF elimination was different *in vitro*. This finding suggests that CAFs and cancer cells are interdependent, sharing a common fate for survival. We propose that this interdependence may stem from the requirement of direct or indirect support from cancer cells for the survival of CAFs or could be facilitated by bidirectional communication between CAFs and tumor cells. In addition, we cannot discount the possibility that non-specific bystander T-cell activation may be triggered by various factors, rather than the activation of CD3+ T cells through CAF-dependent pathways. Nonetheless, further insights into these mechanisms can be gained through T-cell receptor (TCR) sequencing.

Taken together, despite being derived from a limited set of assays, our findings provide evidence that, mechanistically, the synergistic anti-tumor effects of the high-order combination of OHSV2-DSTE^FAP5/CD3^, CAR-T cells, and immunotoxins, are likely involved in the functions of OHSV2-DSTE^FAP5/CD3^ to prime CD3+ T-cell activation and enhance proliferation of CD4+ and CD8+ T cells and even disrupt the entangled relationship between cancer cells and fibroblasts.

## Discussion

In HCC, cancer cells are embedded within a complex TME composed of diverse non-malignant cell populations, including immune cells, stromal cells, endothelial cells, smooth muscle cells, adipocytes, and neurons. These cells, along with the various growth factors, cytokines, chemokines, kinases, and proteases secreted from them, all together form a highly structured and vascularized TME (29, 30). Given the intricate interactions within this ecosystem, the TME inevitably influences the efficacy of immunotherapies and contributes to the development of therapeutic resistance, which is characterized by interwoven and overlapped profiles in multiple aspects (31).

One of the predominant challenges of resistance lies in the quality and quantity of antigens, which determines the immunogenicity of HCC cells. In addition, numerous obstacles hinder the recruitment of effector T cells into tumors and the formation of tertiary lymphoid structures (TLS)—organized lymphoid aggregates containing CD4+ T cells, CD8+ T cells, and CD20+ B cells—which have been observed in HCC and other malignancies. Preclinical and clinical studies have provided substantial evidence supporting these immunological hurdles (31, 32). On the contrary, without doubt, immune-suppressive cells, such as Tregs, tumor-associated macrophages (TAMs), and MDSCs, which maintain immune balance in the host’s homeostasis under normal physiological conditions, can naturally counter effector T cells or are hijacked by cancer cells in the TME to suppress T-cell trafficking, proliferation, and effector function. Alternatively, several stromal cells, especially CAFs, can undergo reprogramming to promote immune evasion and enhance cancer cell survival, leading to resistance to immunotherapies (33). Lastly, metabolic crosstalk between immune cells and cancer cells within the TME can also drive immunotherapeutic resistance by altering nutrient availability and immune cell functionality.

Therefore, expanding the arsenal of HCC treatments to overcome drug resistance represents a theoretically sound approach. Similarly, exploring finely tuned combination strategies is realistic due to emerging insights from clinical trials that combination therapy for HCC yields a better prognosis (3, 34).

OVs have emerged as promising therapeutic agents (35); however, their clinical efficacy as single-agent therapy is far from satisfactory in poorly immunogenic cancers, like HCC (6, 36). One of the putative challenges is the tumor stroma, like CAFs, which can prevent effective dissemination from OVs even following intratumoral injection. Hence, targeting CAFs presents an ideal strategy to enhance antitumor efficacy, as they are among the most abundant stromal components in the tumor microenvironment (TME) and exhibit minimal mutational evolution, reducing the likelihood of acquired drug resistance (20, 37–41). Indeed, our constructed OHSV2-DSTE^FAP/CD3^ expressing DSTEs and an adenovirus ICO15K-FBiTE targeting FAP on CAFs both can exert superimposed effects through simultaneously inducing cancer cell lysis by OVs and fostering immune synapse-mediated depletion of FAP+ CAFs by CD3+ T cells (40, 42), thus outperforming their parental virus alone. Another aim of combining OVs with DSTEs is to balance antiviral and antitumor immunity by redirecting T cells to kill CAFs rather than clearing OVs because the total number of T cells is limited, especially in the TME. However, our OHSV2-DSTE^FAP/CD3^ alone cannot eliminate CAFs to reduce the density of the extracellular matrix *in vivo* experiments, compared to that *in vitro*, where both various stakeholders and our DSTEs have great opportunities to closely contact, or intertwine with each other. Thus, while CAF targeting is conceptually appealing, our data suggest that monotherapeutic blocking CAFs may not be a perfect strategy, at least for HCC treatment *in vivo*, despite attractiveness. For this reason, we considered exploring new strategies, although whether a path ahead is hidden by towering mountains remains uncertain.

CAR-T cells have yet to achieve comparable success against solid tumors up to the present, although representing a revolutionary immunotherapy in B-cell-related hematological cancers. Potentially challenging issues have been highlighted, including low specificity and high heterogeneity of target antigens (increasing the risk of on-target/off-tumor toxicity), inadequate trafficking and persistence, and suboptimal effector function (17). While nearly 100 innovative therapeutic strategies are currently emerging in CAR-T cell development pipelines, combinatorial approaches remain the most promising avenue to improve CAR-T cell infiltration, proliferation, and functional persistence (43). Ovs represent a leading candidate for combination therapy, since they cannot only induce immunogenic cell death by releasing soluble tumor-derived antigens and danger-associated molecular patterns (DAMPs) but also exhibit favorable safety profiles when combined with other cancer treatment approaches with alternative mechanisms (44). Antibody–drug conjugates (ADCs) are promising cancer treatment modalities through selective delivery of highly cytotoxic payloads to tumors; however, the challenges remain evident, such as the lack of highly specific and internalizable antigens and payloads with low off-target toxicity. Alternatively, immunotoxins, like our J80A-PE24, which is derived from anti-GPC3 antibody fragments conjugated to PE24, possess high specificity and low toxicity, yet are accompanied by their insufficient standalone anti-tumor effect (27).

Indeed, our data demonstrated that the high-order combination of OHSV2-DSTE^FAP/CD3^, J80A-PE24, and CAR-T^HN3^ can induce an utmost significant tumor regression and prolonged survival compared to the others, such as angiogenesis inhibition and PD-1 blockade. Meantime, we found that OHSV2-DSTE^FAP/CD3^-based therapy exhibits the absence of off-tumor toxicity, including cytokine storm, inconsistent with previous studies on FAP targeting therapy and CAR-T therapy, despite GPC3 and FAP are suboptimal targets owing to their low specificity (45, 46). Such findings could be explained by the intratumoral injection and selective replication of Ovs in cancer cells and the short half-life of DSTEs in serum and an appropriately optimized dosage, as supported by circulating cytokine levels and tissue histopathology analyses. Additionally, our expectation of selecting CAR-T^HN3^ and J80A-PE24 specifically targeting GPC3 (15, 27) was to avoid coordination challenges in later-stage development, considering the scarcity of successful immunotherapy combinations involving agents from different pharmaceutical sponsors. However, it should be noted that our data could not definitively establish the superiority of OHSV2-DSTE^FAP/CD3^ over PD-1 inhibitors, since we did not directly compare them as monotherapies. Finally, OHSV2-DSTE^FAP/CD3^-based combination therapy could potentially augment the vulnerability of tumors by fostering a hot TME, as evidenced by the heightened infiltration of CD4+ and CD8+ T cells and amplification of exhaustion-related genes. Thus, these findings lay the groundwork for future combination strategies involving ICIs, aligning with our previous research outcomes (10, 47). T-cell exhaustion is crucial for immunotherapy, as overall, antitumor immunity would not manifest in its absence. Our previous research found that oncolytic virus OHSV2 treatment significantly reduces T-cell exhaustion markers on the cell surface, such as CTLA-4, TIM3, LAG3, and TIGIT (48). Similarly, immunotoxin treatment has been shown to activate the antitumor activity of immune cells (49). Bispecific antibodies directly activate T cells, and previous studies have also shown that T-cell exhaustion correlates with drug resistance (50, 51). Regarding CAR-T cell therapy, the situation may be more complex because exhaustion of the CAR-T cells themselves may reduce immune effector function, and it remains unclear whether they affect the activation and subsequent exhaustion of circulating or tissue-resident T cells within the host per se. Thus, further in-depth investigation of key T-cell exhaustion-related markers in this high-order combination therapy is warranted, especially after clinical application, to confirm their predictive efficiency for therapeutic outcomes.

The effectiveness of OHSV2-DSTE^FAP/CD3^ in eradicating CAFs is limited when used alone but becomes significantly powerful when combined with other treatments, implying a robust correlation between CAF reduction and tumor regression. Therefore, we speculated that this might be due to the survival of CAFs being supported by redundant signaling pathways and cytokines (such as TGF-β, POSTN, ACTA2, MMP11, TAGLN, and FN1). More importantly, mutual reshaping and interdependence between CAFs or other cells (like Treg cells) and cancer cells were successfully interrupted by our high-order combination therapy. The reason for the inconsistency in *in vitro* results could be that it is challenging to co-culture multiple cell types to accurately mimic the tumor microenvironment *in vitro*. Conversely, *in vivo*, fibroblasts receive additional external signaling inputs from other cell types such as tumor cells, Treg cells, or TAMs. Once a substantial number of tumor cells are eliminated, the suppression of fibroblasts may be further amplified. Logically, this discrepancy primarily reflects that conclusions drawn from *in vitro* studies cannot be simply extrapolated to *in vivo* conditions, much like how animal experiments cannot fully substitute for clinical trials, particularly in the development of immunotherapeutic agents. However, unraveling the underlying mechanism remains complex, as CAFs are considered to not only possess immunosuppressive or tumor-promoting functions but also potentially fuel anti-tumor immune functions in a specific context (52, 53).

Additionally, it is important to acknowledge the significant limitations of our study. First, it is still unclear whether the antitumor effects are predominantly mediated by CAR-T cells or whether all three drugs contribute equivalently. Second, our mechanistic analysis does not fully address the specific roles of CD3+ T cells, CD4+ T cells, and CD8+ T cells in antitumor immunity. Finally, we acknowledge that the subcutaneous model does not fully replicate the complex tumor microenvironment of HCC, particularly with respect to liver-specific stromal interactions and immune cell infiltration. However, in the context of immunotherapy drug development, designing antibodies based on mouse tumor antigens is not suitable for future human studies. Since immunocompetent mouse models possess their own intact immune systems, they are better suited for investigating the impact of the host immune system on immunomodulatory agents, although in our study, we transplanted human PBMCs to fall short of fully mimicking a complete immune system. For instance, they lack other lymphocyte components and are unable to adequately represent the immune responses occurring within the tumor microenvironment, lymph nodes, or other lymphoid structures, and the communication between them. Indeed, in our earlier studies on oncolytic viruses, we employed immunocompetent mouse models for mechanistic analysis. However, to develop drugs intended for future clinical application in humans, our study utilized human bispecific antibodies, immunotoxins, and CAR-T cells. Consequently, if normal immunocompetent mice were used as the model, the treatment would fail to exert inhibitory effects on the engrafted murine tumors. On the contrary, the immune system of mice would mount a robust response against the exogenous antibodies and T cells, leading to severe autoimmune reactions, which would not only undermine the ability to effectively kill tumor cells but could also result in life-threatening toxicities. This explains why many immunotherapeutic agents, despite demonstrating clear antitumor activity and gaining clinical approval, still have unclear mechanisms of efficacy and toxicity. Undoubtedly, our study also falls short in presenting a clear and comprehensive grand spectacle of the interactions between drug efficacy, toxicity, and mechanism, primarily due to the absence of in-depth, model-driven mechanistic investigations.

Moreover, while the absence of one or two additional groups may lead to incomplete mechanistic explanations, we are confident that this limitation does not significantly impact the robustness of our evidence regarding efficacy and safety.

While promising, the path to clinical translation remains fraught with challenges. Specifically, we suggest that this multi-drug combination strategy holds potential for future extension but will require further exploration through additional animal experiments and confirmation in clinical studies. Relative to OVs and immunotoxins, the high cost of CAR-T cell therapy is a significant challenge, primarily due to the expenses associated with personalized manufacturing. In the future, if widely applied in solid tumors, cost-sharing mechanisms among individuals may help mitigate this burden. Additionally, advancements in preparation methods and technologies, such as *in vivo* gene editing and delivery techniques, accessible cell culture technologies in medical centers, or off-the-shelf stem cell preparation technologies, present potential solutions to reduce costs. Finally, the market regulation of CAR-T cell therapy remains challenging to standardize due to its unique personalized features, including factors such as preparation time, cell dosage, cell viability, bridging treatment strategies, and the logistical capabilities of medical centers. Addressing these challenges requires collaborative efforts among researchers, industry stakeholders, and regulatory agencies to develop scalable, affordable, and regulatory-compliant treatment strategies.

In summary, our findings highlight the utility of OHSV2-DSTE^FAP5/CD3^ as a potent biological agent for enhancing local immune responses and suppressing CAFs, albeit their limited impact *in vivo* and reliance on cancer cells. Moreover, compelling evidence from our study underscores the efficacy of a high-order combination therapy with non-overlapping resistance profiles at sub-maximal tolerated doses, leading to substantial tumor regression, including a 40% complete response rate, as a Chinese proverb says: suddenly, another village with green trees and bright flowers comes in sight.

## Conclusions

This study demonstrates that OHSV2-DSTE^FAP5/CD3^ is capable of eradicating CAFs *in vitro* and remodeling the local tumor microenvironment *in vivo*. A proof-of-concept combination therapy involving OHSV2-DSTE^FAP5/CD3^, J80A-PE24, and CAR-T^HN3^ shows promise, with synergistic anti-cancer effects and acceptable safety profiles. Thereby, without a doubt, this innovative approach paves the way for further investigation for translation into clinical applications for the treatment of HCC and potentially other types of cancer.

## Data Availability

The original contributions presented in the study are included in the article/**Supplementary Material**, further inquiries can be directed to the corresponding author/s.

## Supplementary material

Supplementary Figure 1 OHSV2-DSTE^FAP5/CD3^ dose-finding study *in vivo*. **(A)** Tumor growth curves in four OV treatment groups. The 4 groups were treated respectively with OHSV2-DSTE^FAP5/CD3^-E6 (black), OHSV2-DSTE^FAP5/CD3^-E7 (blue), OHSV2-DSTE^FAP5/CD3^-E8 (orange), and OHSV2-GFP (green). HuH-7 tumor cells (3×10^6^) were subcutaneously inoculated into BALB/c nude mouse. Treatment was initiated once tumors reached approximately 100 mm³ (n=5 mice per group). Data were presented as mean ± SD, and two-way ANOVA with Tukey’s multiple comparisons test was performed. ns, not significant; ***, *p*<0.001. **(B)** Levels of cytokines *in vivo*, including IFN-γ, IL-6, IL-2, IL-4, IL-10, and TNF.

Supplementary Figure 2 Single-cell RNA sequencing analysis of tumor tissues. **(A)** Seven cell clusters identified from tumor tissues by single-cell RNA sequencing, including B cells, CD3+CD4+T cells, CD3+CD8+T cells, iCAF, myCAF, TAMs, and TECs. CAFs, cancer-associated fibroblasts; myCAF, myofibroblastic CAFs; and iCAF, inflammatory CAFs. **(B)** UMPA plot of different lymphocytes and CAFs derived from tumor tissues of all groups. **(C)** Violin plot illustrating the expressions of iCAF and myCAF in their respective marker genes.

Supplementary Table 1 Partial parameters reflecting momentous organ system function. Seven groups are as follows: Group 1, control group; Group 2, J80A-P24; Group 3, Lenvatinab + anti-PD-1; Group 4, OHSV2-DSTE^FAP5/CD3^; Group 5, OHSV2-DSTE^FAP5/CD3^ + J80A-PE24; Group 6, OHSV2-DSTE^FAP5/CD3^ + J80A-P24 + anti-PD-1; and Group 7, OHSV2-DSTE^FAP5/CD3^ + J80A-PE24 + CAR-T^HN3^. WBC, white blood cells; RBC, red blood cells; TBIL, total bilirubin; ALT, glutamate transaminase; CK-MB, phosphocreatine kinase; and Cr, creatinine.

Supplementary Table 2 Distribution of cell numbers of CD3+CD4 + T and CD3+CD8+ T cells in different treatment groups.
