# Supplementary figures and images for: A conceptual exploration on the synergistic anti-tumor effects of high-order combination of OHSV2-DSTEFAP5/CD3, CAR-T cells, and immunotoxins in hepatocellular carcinoma
Source: Front Immunol. 2025 May 8;16:1509087. doi: 10.3389/fimmu.2025.1509087 (PMC12095149; doi:10.3389/fimmu.2025.1509087)

Supplementary fig 1

A

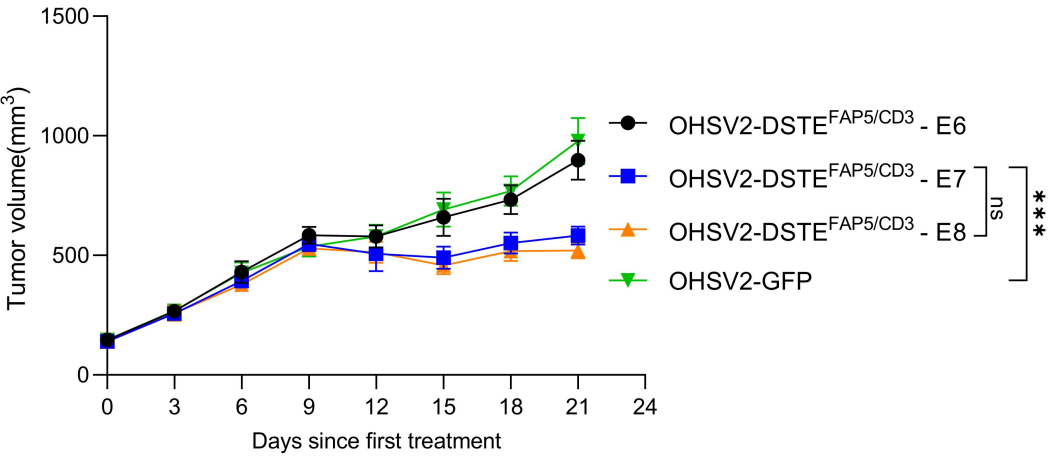

B

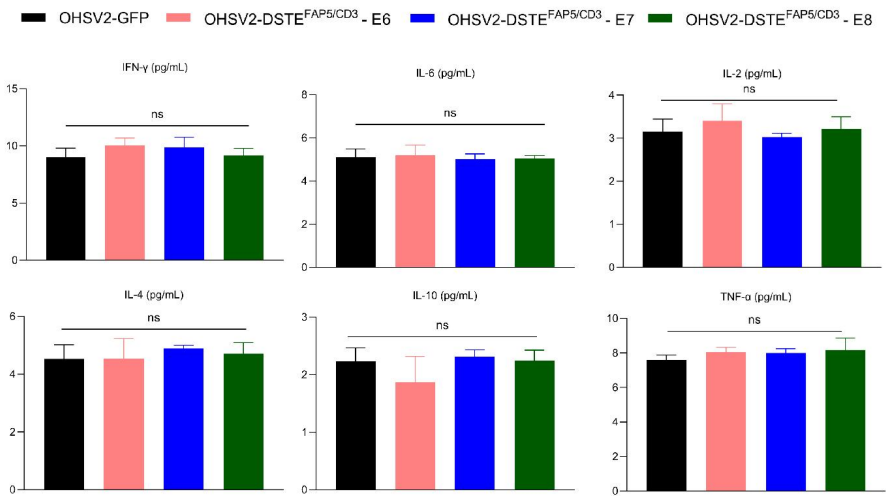

Supplement: Supplementary Figure 1 — OHSV2-DSTEFAP5/CD3 dose-finding study in vivo. (A) Tumor growth curves in four OV treatment groups. The 4 groups were treated respectively with OHSV2-DSTEFAP5/CD3-E6 (black), OHSV2-DSTEFAP5/CD3-E7 (blue), OHSV2-DSTEFAP5/CD3-E8 (orange), and OHSV2-GFP (green). HuH-7 tumor cells (3×10^6^) were subcutaneously inoculated into BALB/c nude mouse. Treatment was initiated once tumors reached approximately 100 mm³ (n=5 mice per group). Data were presented as mean ± SD, and two-way ANOVA with Tukey’s multiple comparisons test was performed. ns, not significant; ***, p<0.001. (B) Levels of cytokines in vivo, including IFN-γ, IL-6, IL-2, IL-4, IL-10, and TNF. [file Image1.pdf]

# Supplementary fig 2

A

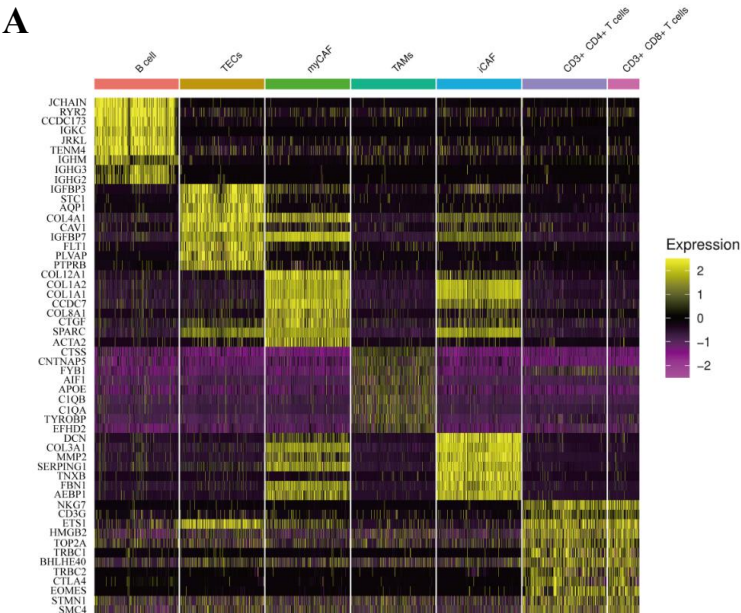

B

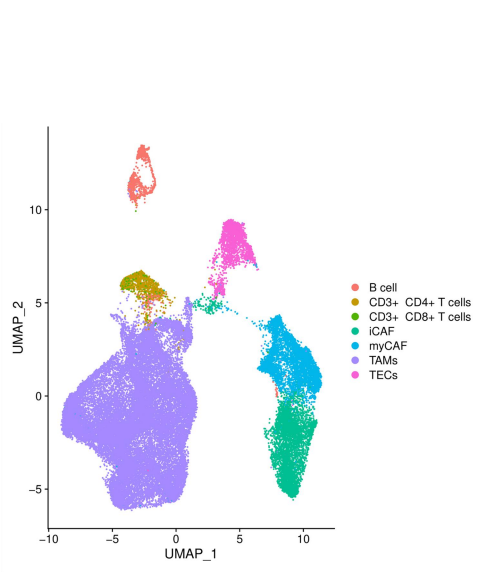

C

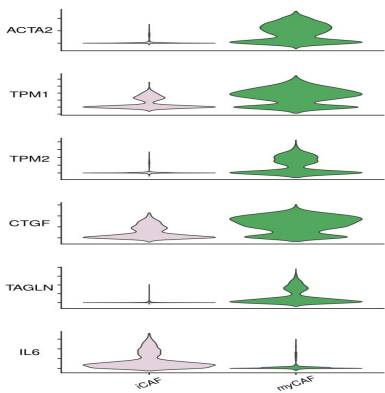

Supplement: Supplementary Figure 2 — Single-cell RNA sequencing analysis of tumor tissues. (A) Seven cell clusters identified from tumor tissues by single-cell RNA sequencing, including B cells, CD3+CD4+T cells, CD3+CD8+T cells, iCAF, myCAF, TAMs, and TECs. CAFs, cancer-associated fibroblasts; myCAF, myofibroblastic CAFs; and iCAF, inflammatory CAFs. (B) UMPA plot of different lymphocytes and CAFs derived from tumor tissues of all groups. (C) Violin plot illustrating the expressions of iCAF and myCAF in their respective marker genes. [file Image2.pdf]
